# Supplementary figures and images for: Transcriptome and metabolome response of eggplant against Ralstonia solanacearum infection
Source: PeerJ. 2023 Jan 11;11:e14658. doi: 10.7717/peerj.14658 (PMC9840387; doi:10.7717/peerj.14658)

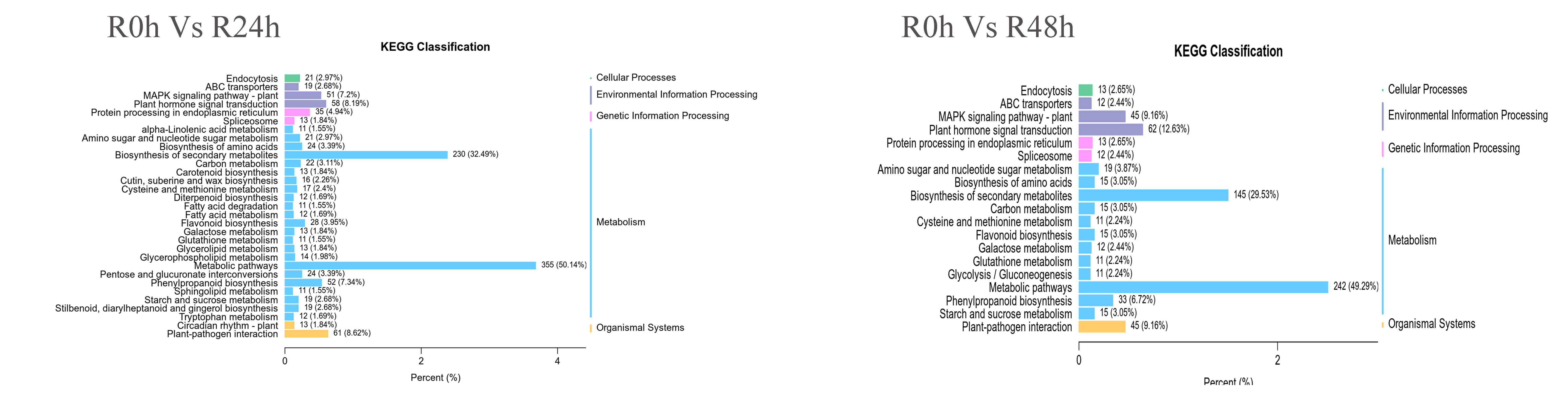

Supplement: Supplemental Information 1 [file peerj-11-14658-s001.jpg]

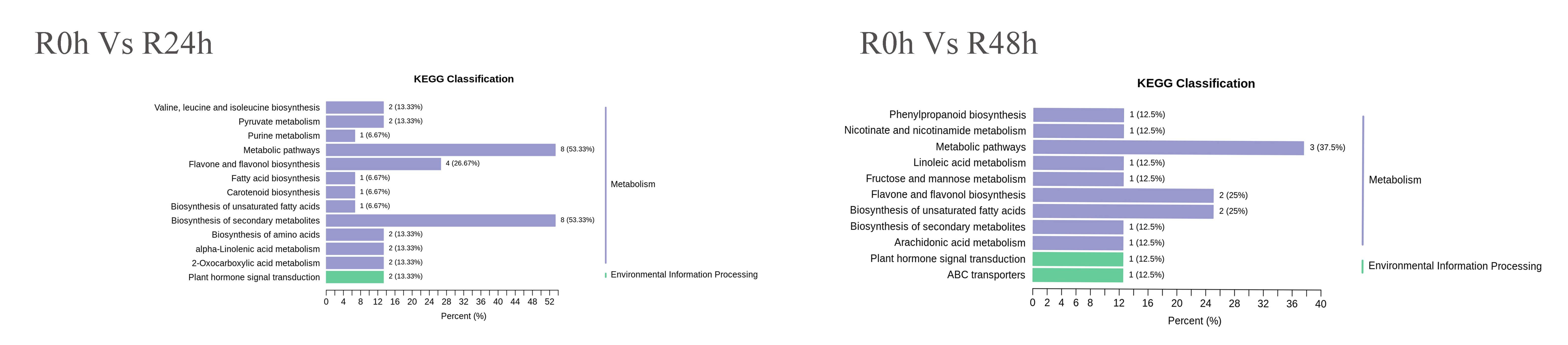

Supplement: Supplemental Information 2 [file peerj-11-14658-s002.jpg]

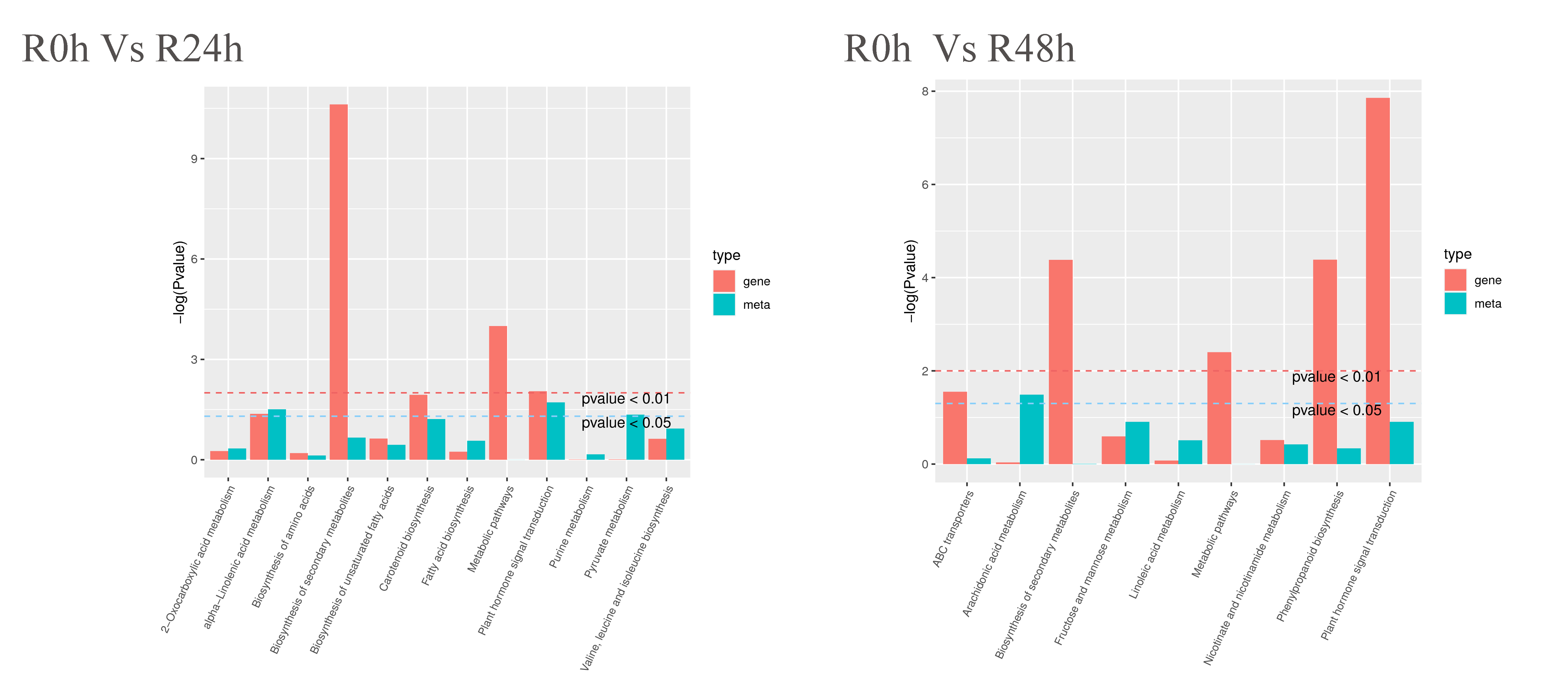

Supplement: Supplemental Information 3 [file peerj-11-14658-s003.jpg]
