# Supplementary material for: Transcriptome and metabolome response of eggplant against Ralstonia solanacearum infection
Source: PeerJ. 2023 Jan 11;11:e14658. doi: 10.7717/peerj.14658 (PMC9840387; doi:10.7717/peerj.14658)
Supplement: Supplemental Information 5 [file peerj-11-14658-s005.docx]

Table S4 The DEGs were selected as absolute Log2 >5 after the eggplant inoculation with *R.sonalacearum*

|  | Gene ID | Log2Fold | NR annotation |
| --- | --- | --- | --- |
| R0h Vs R24h | SMEL_001g129420.1 | -5.919 | PREDICTED: acidic endochitinase-like [Solanum tuberosum] |
|  | SMEL_002g154110.1 | 5.408 | Cytochrome c biogenesis CcmF C-terminal-like mitochondrial protein [Capsicum chinense] |
|  | SMEL_002g161940.1 | 5.513 | hypothetical protein M569_00222, partial [Genlisea aurea] |
|  | SMEL_003g171730.1 | -5.048 | PREDICTED: protein IQ-DOMAIN 1-like [Nicotiana tabacum] |
|  | SMEL_006g242810.1 | 5.472 | PREDICTED: receptor-like protein 12 [Solanum tuberosum] |
|  | SMEL_006g247430.1 | -6.097 | PREDICTED: UDP-glucose iridoid glucosyltransferase-like [Nicotiana tabacum] |
|  | SMEL_006g265100.1 | 5.424 | PREDICTED: protein DOWNY MILDEW RESISTANCE 6-like [Capsicum annuum] |
|  | SMEL_008g308240.1 | -5.056 | PREDICTED: probable ascorbate-specific transmembrane electron transporter 1 [Nicotiana tomentosiformis] |
|  | SMEL_008g311140.1 | -5.529 | PREDICTED: uncharacterized protein LOC101253513 [Solanum lycopersicum] |
|  | SMEL_008g314550.1 | -5.027 | PREDICTED: 17.6 kDa class I heat shock protein [Solanum tuberosum] |
|  | SMEL_009g331050.1 | 5.824 | PREDICTED: late embryogenesis abundant protein D-34-like isoform X1 [Solanum lycopersicum] |
|  | SMEL_010g352490.1 | 5.293 | PREDICTED: leucoanthocyanidin dioxygenase-like [Solanum tuberosum] |
|  | SMEL_010g354680.1 | 5.502 | PREDICTED: protein SRG1-like [Solanum tuberosum] |
|  | SMEL_011g365010.1 | -5.642 | PREDICTED: uncharacterized serine-rich protein C215.13-like [Solanum pennellii] |
|  | SMEL_011g378660.1 | 5.137 | PREDICTED: ethylene-responsive transcription factor ABR1-like [Solanum tuberosum] |
|  | SMEL_012g385870.1 | -5.205 | PREDICTED: uncharacterized protein LOC104591250 [Nelumbo nucifera] |
|  | SMEL_012g396650.1 | -5.938 | PREDICTED: germin-like protein 9-3 [Solanum tuberosum] |
| R0h Vs 484h | SMEL_003g192780.1 | -5.075 | PREDICTED: putative WEB family protein At4g17210 [Solanum tuberosum] |
|  | SMEL_003g199760.1 | -6.547 | Ethylene-responsive transcription factor LEP [Capsicum annuum] |
|  | SMEL_004g202810.1 | -5.609 | PREDICTED: probable glutathione S-transferase [Solanum tuberosum] |
|  | SMEL_007g288370.1 | -5.216 | hypothetical protein CQW23_17197 [Capsicum baccatum] |
|  | SMEL_008g305190.1 | -5.244 | PREDICTED: serine/threonine-protein kinase STY17-like [Solanum pennellii] |
|  | SMEL_008g311140.1 | -5.428 | PREDICTED: uncharacterized protein LOC101253513 [Solanum lycopersicum] |
|  | SMEL_008g314940.1 | -6.371 | PREDICTED: phospholipase D C-like [Solanum tuberosum] |
|  | SMEL_010g336730.1 | -5.857 | PREDICTED: palmitoyl-acyl carrier protein thioesterase, chloroplastic-like [Solanum pennellii] |
|  | SMEL_010g349430.1 | -6.332 | Glutathione S-transferase U18 [Capsicum baccatum] |
|  | SMEL_010g349720.1 | -5.489 | hypothetical protein BC332_27939 [Capsicum chinense] |
|  | SMEL_011g367170.1 | -5.406 | PREDICTED: probable serine/threonine-protein kinase At1g09600 [Solanum pennellii] |
| R24h Vs R48h | SMEL_010g352490.1 | -5.307 | PREDICTED: leucoanthocyanidin dioxygenase-like [Solanum tuberosum] |
